# Prevalence and Impact of Rheumatologic Pain in Cystic Fibrosis Adult Patients

**DOI:** 10.3389/fmed.2021.804892

**Published:** 2022-02-08

**Authors:** Axelle Schmoll, Claire Launois, Jeanne-Marie Perotin, Bruno Ravoninjatovo, Muriel Griffon, Sophie Carré, Pauline Mulette, Julien Ancel, Jean Hagenburg, François Lebargy, Gaëtan Deslée, Jean-Hugues Salmon, Sandra Dury

**Affiliations:** ^1^Department of Respiratory Diseases, Reims University Hospital, Reims, France; ^2^INSERM UMRS 1250, University of Reims Champagne-Ardenne, Reims, France; ^3^EA7509 IRMAIC, University of Reims Champagne-Ardenne, Reims, France; ^4^Department of Rheumatology, Reims University Hospital, Reims, France; ^5^EA3797, University of Reims Champagne-Ardenne, Reims, France

**Keywords:** cystic fibrosis, pain, joint, spinal, rheumatologic, quality of life

## Abstract

**Background:**

With the improvement of cystic fibrosis (CF) patient survival, the prevalence of long-term complications increased, among them rheumatologic disorders.

**Methods:**

The aim of this prospective study was to evaluate the prevalence of spinal and joint pain, and their impact on disability, anxiety, depression, and quality of life in CF adult patients.

**Results:**

Forty-seven patients were analyzed, 72% of men, mean aged 28 years, with a mean body mass index of 22 kg/m^2^ and a mean FEV_1_% of 63%. Twenty-two patients (47%) described rheumatologic pain either spinal (*n* = 15, 32%) and/or joint pain (*n* = 14, 30%). Patients with spinal and/or joint pain were shorter (*p* = 0.023), more frequently colonized with *Staphylococcus aureus* (*p* < 0.008), had more frequent ΔF508 homozygous mutations (*p* = 0.014), and a trend for more impairment of the 6-min walking distance (*p* = 0.050). The presence of rheumatologic pain tended to be associated with disability according to the Health Assessment Questionnaire (HAQ) and anxiety. Compared with patients with no pain patients with both spinal and joint pain exhibited a more pronounced impact on the St George's Respiratory Questionnaire (SGRQ).

**Conclusion:**

Rheumatologic pain is frequent in CF adult patients, and may affect daily living, anxiety and quality of life. Systematic assessment of rheumatologic pain should be included in the management of CF patients.

## Background

Cystic fibrosis (CF) is the most common life-threatening genetic disease in Caucasian populations. Morbidity and mortality depend mainly on chronic respiratory failure and malnutrition. Beside usual clinical and spirometrics parameters and sweat chloride concentrations, emergent tools including biomarkers measured in blood, sputum or bronchoalveolar lavage (1), rheologic tests and low field nuclear magnetic resonance (2) may help to evaluate disease prognosis and efficacy of new pharmological treatments in the future.

With improving survival, the prevalence of long-term complications increased (3). In CF adults, rheumatologic disorders are frequently encountered (4, 5) including CF arthropathy (CFA) (2–29%) (6–9), CF related bone disease (CFBD) including osteoporosis, fractures, and musculoskeletal manifestations (13 to 35%) (10–13), hypertrophic pulmonary osteoarthropathy (2 to 7%) and quinolone-induced arthropathy (14).

The definition of CFA is not fully accepted. Clinical manifestations are heterogeneous, usually characterized by recurrent transient episodes of painful mono- or polyarthritis lasting 1 day to several weeks and not classifiable as any of other rheumatic diseases (4, 7, 8, 14, 15). Joints of hands, feet, and knees are the most frequent sites (6, 8). The pathogenesis is unknown and its associations with respiratory exacerbations appear uncertain (4, 15). Intermittent arthritis may become chronic over time (8).

Pain is a common symptom in CF that impacts the quality of life (16–18), mood, work (17), and clinical outcomes (17). In adults, one of the most frequent pain locations is rheumatologic sites including back (15–70%), bone or muscles (44%), and joints (5–41% depending on the sites) (16–19). So far, no study specifically assessed the impact of rheumatologic pain on the quality of life in CF.

The aim of our study was to determine the prevalence of rheumatologic pain (spinal and/or joint) in a cohort of CF adult patients. In addition, we evaluated the impact of pain on patient's disability (from the Health Assessment Questionnaire widely used in rheumatic diseases for evaluating dependence), anxiety and depression (from the Hospital Anxiety and Depression Scale), and quality of life by Cystic Fibrosis Questionnaire for teenagers and adults (CFQ 14+), St George's Respiratory Questionnaire (SGRQ) and Medical Outcome Study Short Form 36 health survey (MOS SF-36) usually used in CF studies.

## Materials and Methods

### Study Design

This monocentric study was prospectively conducted in the Department of Respiratory Diseases (University Hospital of Reims) between November 2016 and December 2019. Patients were included in the RINNOPARI study (Recherche et INNOvation en PAthologie Respiratoire Inflammatoire), an observational cohort of inflammatory chronic lung diseases. The study was approved by the Ethics Committee of Dijon EST I on 31st May 2016 (N°2016-A00242-49) and by the French National Agency for Medicines and Health Products (ANSM) on 25th April 2016. The protocol was registered on ClinicalTrials.gov (NCT02924818) on 5th October 2016. Each patient signed a written informed consent form.

CF patients were included if they were at least 18 years of age. Exclusion criteria were previous or planned lung transplantation and patients requiring an urgent visit. Anonymized data including demography, clinical characteristics, pulmonary function tests, and sputum microbiology were collected and registered on an electronic medical record.

Chronic infection by *Pseudomonas aeruginosa* and by extension chronic infection by *Staphylococcus aureus* was defined according to Leeds criteria (20).

### Rheumatologic Assessment

Patients were asked to answer 4 questions regarding rheumatologic symptoms. Two questions assessed rheumatologic pain: one question assessed spinal pain (“Did you have spinal pain?”), and one question assessed joint pain (“Did you have joint pain?”). Patients were classified as either “no pain” (answering “no” to the two questions) or “pain” (answering “yes” to at least one question). Patients with “pain” were classified as “both pain” (spinal and joint pain) or “isolated pain” (spinal pain or joint pain).

Two additional questions assessed arthritis (“Did you have swelling joints? Did you have morning joint stiffness more than 30 minutes?”). Arthralgia associated with swelling joints and/or morning joint stiffness suggested inflammatory joint pain.

Patients' functional disability in the past week was assessed by the Health Assessment Questionnaire (HAQ), a validated scale consisting of eight sections: dressing, arising, eating, walking, hygiene, reach, grip and activities. The final score ranged between 0 (no assistance) and 3 (patient usually needs both a special device and help from another person) (21, 22).

### Symptoms Score and Quality of Life Scales

Anxiety and depression were assessed by the Hospital Anxiety and Depression Scale (HAD) (23, 24).

The quality of life of CF patients was evaluated using dedicated questionnaires: (1) the Cystic Fibrosis Questionnaire for teenagers and adults (CFQ 14+) assessing the quality of life, symptoms, and disease effects. The score ranges from 0 to 100, the highest score corresponding to a better quality of life (25); (2) the St George's Respiratory Questionnaire (SGRQ) assessing symptoms and their impact on everyday activities. The total score includes the sum of 3 domains: impact, activity, and symptoms. A score of 100 indicates maximum impairment of quality of life (26); (3) the Medical Outcome Study Short Form 36 health survey (MOS SF-36) a multifaceted and generic scale assessing health status regardless of causal disease, sex, age, and treatment. A score of 100 indicates no impairment of quality of life (27).

### Study Endpoints

The primary endpoint was the prevalence of rheumatologic pain (spinal and/or joint). Secondary endpoints were to evaluate the impact of rheumatologic pain on the patient's functional disability, mental health, and quality of life (HAQ, HAD, CFQ 14+, MOS SF-36, and SGRQ).

### Statistical Analysis

Data were described as numbers (percentages), mean ± standard deviation. Given the limited number of patients, differences in all variables were assessed using Fisher's exact tests for qualitative variables and Mann–Whitney U-tests for quantitative variables. A correction was applied for multiple comparisons according to the Benjamini Hochberg procedure. A *p*-value <0.05 was considered statistically significant. Results were analyzed with SPSSv27.

The Cronbach's alpha value for each quality of life scores (HAQ, HAD, CFQ 14+, SGRQ and MOS SF-36) was calculated. A value > 0.7 was considered as a high level of consistency.

## Results

Fifty-one consecutive CF patients were included in the study. Four patients were excluded (*n* = 3 for clinical rheumatologic missing data; *n* = 1 for previous lung transplantation), 47 patients were analyzed.

### Patient Characteristics and Rheumatologic Assessment

Demographic, clinical, bacteriological characteristics, and rheumatologic assessment of patients are detailed in Table 1. Seventy-two percent of patients were men. The mean age was 28 years with a mean body mass index of 22 kg/m^2^. Mean FEV_1_% was 63% of the predicted value. The mean distance on the 6-min walking test was 80 ± 11% of the predicted value. Main treatments are detailed in Supplementary Table S1. Painkillers included paracetamol (*n* = 4, 8%), tramadol (*n* =1 , 2%) and non-steroidal anti-inflammatory drugs (*n* = 2, 4%).

**Table 1:** Clinical and rheumatologic characteristics, lung function, and microbiology data.

	**Total**	**No pain**	**Pain**	***p*-value**
*n*	47	25 (53)	22 (47)	
Male	34 (72)	20 (80)	14 (64)	0.211
Age, years	28 ± 9	30 ± 10	26 ± 8	0.121
Height, cm	170 ± 8	172 ± 9	167 ± 7	0.023
Weight, kg	63 ± 12	65 ± 14	60 ± 11	0.228
BMI, kg/m^2^	22 ± 3	22 ± 3	22 ± 3	0.773
CFTR mutation				
ΔF508 homozygous	19 (40)	6 (24)	13 (59)	0.014
ΔF508 heterozygous	24 (51)	16 (64)	8 (36)	0.059
Other	4 (8)	3 (12)	1 (4)	0.611
Pancreatic insufficiency	37 (79)	18 (72)	19 (86)	0.230
Diabetes	16 (34)	7 (28)	9 (41)	0.351
Depression	7 (15)	4 (16)	3 (14)	0.820
Osteoporosis	9 (22)	4 (20)	5 (28)	0.573
Rheumatologic symptoms				
Spinal pain	15 (32)	0	15 (68)	–
Joint's pain	14 (30)	0	14 (64)	–
Swelling joints	1 (2)	1 (4)	0	0.343
Morning joint stiffness > 30 min	4 (8)	1 (4)	3 (14)	0.237
Exacerbation in the last year	31 (66)	16 (64)	15 (68)	0.682
Number of episodes per patient	2.3 ± 1.4	2.5 ± 1.5	2.2 ± 1.3	0.561
Chronic bacterial colonization				
*Pseudomonas aeruginosa*	19 (40)	12 (48)	7 (32)	0.241
*Staphylococcus aureus*	34 (72)	14 (56)	20 (91)	0.008
Spirometry				
FEV_1_, %	63 ± 30	58 ± 28	68 ± 32	0.245
TLC, %	111 ± 13	109 ± 13	115 ± 12	0.255
6-min walking test				
Distance, %	80 ± 11	84 ± 10	74 ± 10	0.050

*Data are expressed as frequency (percentage) or mean ± standard deviation*.

*BMI, body mass index; CFTR, cystic fibrosis transmembrane conductance regulator; FEV_1_, forced expiratory volume at first second; TLC, total lung capacity*.

Twenty-two patients described rheumatologic pain (47%), including spinal (*n* = 15, 32%) and/or joint pain (*n* = 14, 30%); seven patients (15%) reported both spinal and joint pain. Inflammatory joint pain appeared uncommon (*n* = 3, 6%). Of note, there was no difference in terms of painkillers treatment in patients with pain or no pain (Supplementary Table S1).

Patients suffering from spinal and/or joint pain were significantly shorter (167 ± 7 vs. 172 ± 9 cm, *p* = 0.022), had more frequent ΔF508 homozygous mutations (59 vs. 24%, *p* = 0.014), more frequent colonization by *Staphylococcus aureus* (91 vs. 56%, *p* < 0.008), and had a trend to an impaired 6-min walking distance (74 ± 10% vs. 84 ± 10%, *p* = 0.050). The prevalence of rheumatologic symptoms didn't increase with age (data not shown).

### Impact of Rheumatologic Symptoms

The Cronbach's alpha value for HAQ, HAD, SGRQ, CFQ 14+ and MOS SF-36 was of 0.830, 0.816, 0.913, 0.602, and 0.288, respectively. Because of a low internal reliability, the CFQ 14+ and MOS SF-36 were not considered for the final analysis. Measures of rheumatologic HAQ, HAD and SGRQ are shown in Table 2. HAQ tended to be more impaired in patients with spinal and/or joint pain (0.18 ± 0.23 vs. 0.07 ± 0.16, *p* = 0.061). HAQ was impaired in patients with both spinal and joint pain when compared with patients with no pain (0.36 ± 0.28 vs. 0.07 ± 0.16, *p* < 0.001) (Supplementary Table S2).

**Table 2:** Symptoms score and disability, anxiety and depression, and quality of life scales.

	**Total**	**No pain**	**Pain**	***p*-value**
*n*	44^#^	24 (55)^†^	20 (45)^†^	
HAQ	0.11 ± 0.20	0.07 ± 0.16	0.18 ± 0.23	0.061
HAD				
Anxiety (mean)	6 ± 4	5 ± 3	7 ± 3	0.058
Depression (mean)	3 ± 4	4 ± 3	5 ± 4	0.292
SGRQ				
Impact	20 ± 16	16 ± 14	24 ± 17	0.075
Activity	34 ± 21	29 ± 19	39 ± 22	0.116
Symptoms	45 ± 21	45 ± 22	44 ± 20	0.846
Total	25 ± 17	22 ± 15	29 ± 19	0.125

*Data are expressed as frequency (percentage) or mean ± standard deviation*.

# *Data are not available for 3 patients*.

† *Data are not available for 1 patient with “no pain” and for 2 patients with “pain”*.

*HAD, Hospital Anxiety and Depression Scale; HAQ, Health Assessment Questionnaire; SGRQ, St George's Respiratory Questionnaire*.

The mean anxiety score assessed by the HAD questionnaire trended to be higher in patients with pain (7 ± 3 vs. 5 ± 3, *p* = 0.058) whereas no difference was observed for depression (Table 2). No difference was observed between patients with both spinal and joint pain, and either no pain, isolated spinal and isolated joint pain for anxiety and depression (Supplementary Table S2).

No impact on quality of life assessed by the SGRQ was found in patients with spinal and/or joint pain when compared with patients with no pain (Table 2).

We next analyzed the symptoms scores and SGRQ in patients with both spinal and joint pain (Supplementary Table S2). Compared with patients with no pain, they had a marked impaired quality of life identified in SGRQ (total score, impact and activity domains). Compared with patients with either spinal or joint pain, no difference was observed.

## Discussion

Our study confirmed that rheumatologic pain is frequent, concerning near half of the adult patients with cystic fibrosis (47%). It included spinal pain (32%), joint pain (30%), with 15% of patients suffering from both spinal and joint pain. Inflammatory joint pain appeared uncommon (6%). For the first time, our study focused on the impact of rheumatologic pain on disability, anxiety and depression, and quality of life.

During the past few years, many studies investigated pain in adult CF patients. The prevalence of painful symptoms varies between 89% in the past week (28), 82–89% during the previous month (16, 17) and 94.1% in the past 2 months (19). Painful episodes concern up to three (16.8%) or four (38.4%) different locations (19). Rheumatologic pain is one of the most frequent sites after headache, sinuses, or chest pain (18, 29, 30). In our study, the prevalence of spinal pain was 32%, in line with previous studies reporting between 10 and 28.4% for cervical pain (16, 19) and 50% for dorsal or back pain (16, 17, 19). The prevalence of back pain in the general young population is about 20% (31). However, no comparative study between young adults with or without CF is available. The causes of back pain are not fully established (30, 32). CF adult patients have a low bone mineral density and a high prevalence of osteoporosis (11, 12). Many studies described orthopedic complications of CFBD such as vertebral deformity (33) and scoliosis (13), rib and vertebral fractures (34). Postural abnormalities have been also reported (35). However, none of these studies described the relation between CFBD or postural abnormalities and pain. Lastly, it might be difficult to differentiate musculoskeletal pain from thoracic pain related to the use of accessory respiratory muscles in dyspneic patients with severe disease (16, 32).

Joint pain concerned 30% of our cohort, similar to the previous reviews reporting arthralgia between 20 and 41.4% (16, 19). In Hayes et al. study, the main arthralgia sites were knee (29.7%), wrist (18.9%), and finger (5%) (17). In our study, signs of inflammatory joint pain were unusual (6%). The comparison with other studies is difficult in the absence of an accepted definition of CFA and with various study designs. These studies described a prevalence of inflammatory joint included between 2 and 29% of the patients (6–9, 28). In Koch et al. study there was no significant difference regarding inflammatory signs such as joint swelling or warming between CF patients and controls (6).

Interestingly in our study, patients with spinal and/or joint pain were significantly shorter (167 ± 7 vs. 172 ± 9 cm, *p* = 0.023) and more frequently colonized with *Staphylococcus aureus* (91% vs. 56%, *p* < 0.008). The comparison is difficult with other specific studies concerning musculoskeletal and arthropathy in CF (6, 13, 36, 37). In Roehmel et al. study involving 186 CF children and adults (mean age: 27 years), patients with CFA (defined as at least one symptom out of the following: joint pain, joint swelling, joint reddening or limitation of movement) were more likely to be older, female gender, and to have a higher rate of total IgG, chronic colonization with *Aspergillus* spp. and pulmonary exacerbations (7). In Grehn et al. study from the German CF registry, CFA including arthropathy and arthritis was associated with increasing age, female gender, number of hospitalizations, chronic *Pseudomonas aeruginosa* infection, CF-related diabetes, pancreatic insufficiency and sinusitis/polyps (9). These results also support a correlation between pulmonary inflammation/infection and CFA. We also reported that patients suffering from spinal and/or joint pain had significantly more frequent ΔF508 homozygous mutations (59 vs. 24%, *p* = 0.014). By contrast, two previous studies didn't find an association between cystic fibrosis transmembrane conductance regulator (CFTR) mutations and CFA (7, 9).

From our results, rheumatologic pain may impact daily life activities. First, the 6-min walking distance of painful patients tended to be lower (74 ± 10% vs. 84 ± 10%, *p* = 0.050). To our knowledge, no previous rheumatologic CF study assessed the 6-min walk distance. Second, our results suggest that patients with both spinal and joint pain had a more important impairment on HAQ scale. Their functional disability is probably more important to those of the young general population (38). Some investigators have suggested that the Minimal Clinical Important Difference is 0.1 (39). Only one previous study showed that CF patients reported an impairment in everyday life functions assessed by the HAQ (6). Lastly, the results highlight that patients with both spinal and joint pain had a significant impairment of quality of life according to the SGRQ scores compared with patients with no rheumatologic pain. The majority of chronic diseases worsen health and affect the quality of life (40). Then it is not surprising that rheumatologic pain in CF, a disease including multimorbidity, impacts quality of life. The negative effects of back pain had been previously reported especially on the respiratory and emotion subscale (17). In our study, rheumatologic pain tended to be associated with anxiety but not with depression. Of note, in Hayes et al. study, back pain was also associated with anxiety but not with depression (17). Surprisingly, despite frequent rheumatologic pain in CF patients in our study, very few patients used painkillers, suggesting that rheumatologic pain treatment is overlooked.

There are several limitations to our study. First, our sample size is relatively small which could limit the identification of differences between different groups (painful and not painful patients, patients with spinal, joint and both pain). Second, we have not assessed the consequences of rheumatologic pain on asthenia, sleeping disorders, family life and study or work absenteeism. Third, the four questions used to detect rheumatologic pain do not fully cover the characteristics of pain (acute or chronic, sites, intensity, duration). Lastly, the absence of additional tests for this study carried out in current practice not allowed to identify the origin of pain. A larger study should be conducted to elucidate the potential mechanisms of rheumatologic pain and its therapeutic management.

## Conclusion

Our study confirms that rheumatologic pain is frequent concerning near half of cystic fibrosis adult patients. Patients with spinal and/or joint pain were more frequently colonized with *Staphylococcus aureus* and had more frequent ΔF508 homozygous mutations. The prevalence of rheumatologic symptoms didn't increase with age. No study had previously specifically assessed the impact of rheumatologic pain on patient's disability, anxiety and depression, and quality of life. The impact of both spinal and joint pain seems to be more important, in particular on disability and on quality of life, in comparison with patients with no pain. However, there is no evidence for more painkillers rescue. Our results highlight that the health care team should carefully assess patients and undertake additional tests in collaboration with rheumatologists to identify the cause of pain and therapeutic management.

## Data Availability Statement

The raw data supporting the conclusions of this article will be made available by the authors, without undue reservation.

## Ethics Statement

The RINNOPARI (Recherche et INNOvation en PAthologie Respiratoire Inflammatoire) study was approved by the Ethics Committee of Dijon EST I on 31st May 2016 (No. 2016-A00242-49) and by the French National Agency for Medicines and Health Products (ANSM) on 25th April 2016, and declared on ClinicalTrials.gov (NCT02924818) on 5th October 2016. The patients/participants provided their written informed consent to participate in this study.

## Author Contributions

AS, CL, BR, GD, J-HS, and SD: substantial contributions to the conception. AS, CL, BR, FL, GD, J-HS, and SD: design of the work. AS, J-MP, BR, MG, SC, PM, JA, JH, J-HS, and SD: acquisition and analysis of the data. J-MP: software. AS, CL, J-MP, BR, MG, SC, PM, JA, JH, FL, GD, J-HS, and SD: drafting the work or substantively revising the manuscript. All authors contributed to the article and approved the submitted version.

## Funding

This work was supported by the Reims University Hospital and Champagne Ardennes University (Hospital-University Project named RINNOPARI).

## Conflict of Interest

The authors declare that the research was conducted in the absence of any commercial or financial relationships that could be construed as a potential conflict of interest.

## Publisher's Note

All claims expressed in this article are solely those of the authors and do not necessarily represent those of their affiliated organizations, or those of the publisher, the editors and the reviewers. Any product that may be evaluated in this article, or claim that may be made by its manufacturer, is not guaranteed or endorsed by the publisher.

## Abbreviations

BMI: Body mass index
CF: Cystic fibrosis
CFA: Cystic fibrosis arthropathy
CFBD: Cystic fibrosis related bone disease
CFTR: Cystic fibrosis transmembrane conductance regulator
CFQ 14+: Cystic Fibrosis Questionnaire for teenagers and adults
FEV_1_: Forced expiratory volume at first second
HAQ: Health assessment questionnaire
MOS SF-36: Medical outcome study short form 36 health survey
SGRQ: St George's Respiratory Questionnaire
TLC: Total lung capacity.
