# Supplementary material for: Prevalence and Impact of Rheumatologic Pain in Cystic Fibrosis Adult Patients
Source: Front Med (Lausanne). 2022 Feb 8;8:804892. doi: 10.3389/fmed.2021.804892 (PMC8861186; doi:10.3389/fmed.2021.804892)
Supplement: Supplementary file 1 [file Table_1.docx]

**Table S1 – Therapeutic management of the patients**

|  | Total | No pain | Pain | *p value* |
| --- | --- | --- | --- | --- |
| n | 47 | 25 (53) | 22 (47) |  |
| Inhaler/nebulizer | 36 (77) | 21 (84) | 15 (68) | 0.109 |
| Long-term Azithromycin | 28 (60) | 14 (56) | 14 (64) | 0.672 |
| Physiotherapy | 41 (87) | 21 (84) | 20 (91) | 0.679 |
| Education program | 28 (60) | 15 (84) | 13 (59) | 0.454 |
| Long term oxygen | 2 (4) | 1 (4) | 1 (5) | 0.744 |
| Non invasive ventilation | 2 (4) | 2 (8) | 0 (0) | 0.256 |
| Anxiolytics | 2 (4) | 2 (8) | 0 (0) | 0.360 |
| Antidepressors | 2 (4) | 1 (4) | 1 (5) | 0.640 |
| Painkillers |  |  |  |  |
| Paracetamol | 4 (8) | 1 (4) | 3 (14) | 0.328 |
| Tramadol | 1 (2) | 0 (0) | 1 (5) | 0.468 |
| Non steroidal anti-inflammatory drug | 2 (4) | 0 (0) | 2 (9) | 0.214 |

Data are expressed as frequency (percentage).
