# Supplementary material for: Prevalence and Impact of Rheumatologic Pain in Cystic Fibrosis Adult Patients
Source: Front Med (Lausanne). 2022 Feb 8;8:804892. doi: 10.3389/fmed.2021.804892 (PMC8861186; doi:10.3389/fmed.2021.804892)
Supplement: Supplementary file 2 [file Table_2.docx]

**Table S2 – Disability, anxiety and depression, and quality of life scale according rhumatologic pain**

|  |  |  | Both pain^✝^ | No pain | Spinal pain | Joint pain |
| --- | --- | --- | --- | --- | --- | --- |
| n |  |  | 7 (15) | 25 (53) | 8 (17) | 7 (15) |
| HAQ | | | 0.36 ± 0.28 | 0.07 ± 0.16^#^ | 0.09 ± 0.18 | 0.09 ± 0.11 |
| HAD | | |  |  |  |  |
| Anxiety (mean) | | | 8 ± 3 | 5 ± 3 | 5 ± 2 | 8 ± 4 |
| Depression (mean) | | | 7 ± 4 | 4 ± 4 | 3 ± 3 | 4 ± 3 |
| SGRQ | | |  |  |  |  |
|  | Impact | | 34 ± 19 | 16 ± 14^#^ | 16 ± 10 | 22 ± 19 |
|  | Activity | | 50 ± 18 | 29 ± 19^#^ | 25 ± 17 | 43 ± 26 |
|  | Symptoms | | 63 ± 14 | 45 ± 22 | 44 ± 9 | 28 ± 19 |
|  | Total | | 42 ± 19 | 22 ± 15^#^ | 23 ± 8 | 22 ± 22 |

Data are expressed as mean ± standard deviation.

^✝^ Both pain : spinal and joint pain

^#^ *vs* both pain  : p < 0.05

HAD: Hospital Anxiety and Depression Scale;

HAQ: Health Assessment Questionnaire;

SGRQ: St George’s Respiratory Questionnaire.
